# Preclinical Performance of a Novel Dental Implant Design Reducing Mechanical Stress in Cortical Bone

**DOI:** 10.3390/jfb16030102

**Published:** 2025-03-14

**Authors:** Carolin Erbel, Matthias W. Laschke, Tanja Grobecker-Karl, Matthias Karl

**Affiliations:** 1 Department of Prosthodontics, Saarland University, 66421 Homburg, Germany; carolin.erbel@uks.eu (C.E.); tanja.grobecker-karl@uks.eu (T.G.-K.); 2 Institute for Clinical and Experimental Surgery, Saarland University, 66421 Homburg, Germany; matthias.laschke@uks.eu; 3 Department of Prosthodontics, Philipps University Marburg, 35039 Marburg, Germany

**Keywords:** dental implant, marginal bone loss, primary stability, bone stress

## Abstract

This animal study compared the healing performance of a novel implant design characterized by a shift in thread geometry and core diameter with two different surfaces with that of an apically tapered implant. Test Bioactive (n = 9), Test Porous (n = 7) and Control (n = 8) implants were placed in the mandibles of minipigs. Following healing, bone samples were harvested for determining bone-to-implant contact (BIC) and marginal bone loss (MBL). Comparative statistics were based on Levene’s test, Shapiro–Wilk tests, the Kruskal–Wallis test and Wilcoxon tests with Holm correction (α = 0.05). The mean undersizing of the osteotomy was 0.15 mm for Control, while in the test groups 0.33 mm and 0.34 mm were calculated. Insertion torques ranged from 61.5 Ncm (Control) to 76.1 Ncm (Test Bioactive). Maximum BIC was seen in Test Porous with 55.83%, while Test Bioactive showed only 48.11%. MBL was 4.1 mm in Test Bioactive, while Test Porous and Control exhibited 2.8 mm. No significant differences between the implant groups were observed (*p* > 0.05). Despite greater undersizing, the novel implant type performed comparably to the established Control implants. The rougher surface of the bioactive implants increased the insertion torque and led to more MBL.

## 1. Introduction

Surgical trauma during dental implant placement may cause bone resorption and, hence, esthetic and maintenance problems [1]. Consequently, current implant designs consider minimizing bone trauma and crestal bone loss while maintaining gingival architecture [1]. Thermal and mechanical stress during osteotomy preparation [2], the destruction of bone micro-architecture and the compression of bone tissue [3] for achieving primary implant stability, which itself is governed by undersizing implant and thread design in addition to surface roughness, seem to determine postsurgical peri-implant bone loss. It can be shown that cell death occurs following implant site preparation [4], and consequently, the surgical approach has been claimed to be as important as the implant itself [1].

A vast variety of parameters [2] have been described to govern the temperature increase during drilling, which is a result of friction phenomena between the bone and the drill [5]. These parameters include rotational speed, proceeding speed, contact pressure, drilling motion pattern, bone density [4], irrigation [6,7], drill design, drill fatigue [8], the drill’s material and its heat capacity and thermal conductivity [9] and drill depth [9]. The temperature rise during implant insertion itself also has to be seen as a phenomenon based on friction between the implant body and the bone [5,10,11] governed by implant design, surface roughness, bone quality and the amount of undersizing.

With primary implant stability still being considered a prerequisite for osseointegration, osteotomies are undersized, leading to densification of bone during implant insertion and subsequent microfractures, resulting in a peri-implant zone of dying osteocytes if stress is too pronounced [3,12]. In this context, implant geometry has been shown to have a prevailing effect over surface characteristics on early osseous healing [13]. Based on several studies, the effect of condensing peri-implant bone either during implant site preparation or during implant insertion seems to be negative with respect to healing. Li and coworkers pointed out that condensation caused high interfacial strains and marginal bone resorption but no improvement in implant stability [14], while condensed bone underwent resorption, thereby delaying the onset of new bone formation around the implant [14]. Similarly, excessive osseo-densification has been described to lead to osseo-destruction, resulting in crater-like lesions filled with inflamed granulation tissue [12].

In response to that knowledge, implant manufacturers developed triangular and trioval implant designs, thereby trying not to overstress bone, e.g., through gaining high primary stability by cortical compression [15]. Two clinical studies [16,17], however, did not find major advantages in terms of marginal bone level change as compared to conventionally round implants. On the contrary, a recent retrospective observational study reported a survival rate of only 98% after a mean follow-up time of 1.8 years for an implant with a trioval cross-section and a low-speed site preparation protocol, while the marginal bone level change was −0.53 +/− 1.83 mm from insertion to 1 year [18].

A considerable body of literature exists on how implant design can be utilized for optimizing load transfer to bone [19], with a particular focus on thread geometry [20]. There seems to be a consensus that stress [21] and bone damage [22] do not just induce bone remodeling but may be detrimental to bone integrity [23]. It has been described that primary implant stability is mostly derived from cortical stabilization [24,25], but while this leads to more efficient achievement of secondary stability, high primary stability may be detrimental for maintaining marginal bone levels [26]. Based on these findings, a novel implant design characterized by deep cervical threads and shallow, condensing threads in the middle implant part as well as a shift in implant core diameter was developed [27]. Using an in vitro test setup and employing strain gauge technology, it has been shown that this design reduces mechanical stress in cortical bone during the insertion process while also allowing for primary stability in extraction sites [27,28], as this implant mainly derives primary stability from compressing trabecular bone. Based on these promising findings, it was the goal of the present preclinical study to compare postsurgical bone remodeling of this novel implant with a conventional implant when installed in healed sites.

## 2. Materials and Methods

Following approval by the animal protection committee (Landesamt für Verbraucherschutz des Saarlandes; permission number: 07-2023), this study was conducted in accordance with the Directive 2010/63/EU and the NIH Guidelines for the Care and Use of Laboratory Animals (NIH Publication #85-23 Rev. 1985). A total of five adult (age ranging from 24 months to 30 months; initial weights ranging from 61 kg to 76 kg), female Aachen minipigs [29,30] were allocated for this trial.

Two surgeries were carried out in the mandible of each animal, namely tooth extractions and implant placement. After 12 h of fasting, the animals were sedated with an i.m. injection of ketamine (Ketavet^®^; 30 mg/kg body weight; Zoetis, Berlin, Germany), xylazine (Rompun^®^; 2.5 mg/kg body weight; Bayer Vital GmbH, Leverkusen, Germany) and atropin (Atropinsulfat^®^; 1 mg; B. Braun Melsungen AG, Melsungen, Germany). Subsequently, a venous cannula was placed in the animals’ ears for fluid substitution (0.9% NaCl). Subsequent to endotracheal intubation, general anesthesia was maintained using 2% isoflurane (Portec GME 3^®^; Fritz Stephan GmbH, Gackenbach, Germany), and the animals’ vital parameters (Guardian^®^; RS Meditec Healthcare GmbH, Duisburg, Germany) were permanently monitored. The skin area surrounding the oral cavity was shaved and disinfected using iodine, and the animals were covered by sterile drapes. Each surgery started with an intravenous injection of carprofen (Rimadyl^®^; 10 mg/kg body weight; Zoetis, Berlin, Germany) and a subcutaneous injection of buprenorphine (Bupresol vet.^®^; 0.025 mg/kg body weight; CP Pharma, Burgdorf, Germany) for perioperative analgesia. Furthermore, the single-shot antibiotic ceftiofur (Naxce^®^; 5 mg/kg body weight; Zoetis, Berlin, Germany) was administered through an intramuscular injection. Postoperatively, the animals were kept on a soft diet for ten days.

The third and fourth premolars in addition to the first molar on both sides of the mandible were extracted during the first surgery [2,13]. After a local anesthetic (Ultracain D-S forte^®^; 1:100,000, Sanofi-Aventis, Frankfurt, Germany) was administered, a piezosurgery unit (Piezomed^®^; W&H, Bürmoos, Austria) was employed for tooth cleaning. The multi-rooted teeth were then cut using a red-ring contra angle and carbide burrs. Usual instruments such as forceps and elevators were applied for the extractions, and piezosurgery was available additionally where required. Both interrupted sutures and horizontal mattress sutures (Vicryl^®^ 4-0; Johnson & Johnson Medical, Norderstedt, Germany) were made to approximate the soft tissues and to allow for secondary healing. Intraoral radiographs (Heliodent^®^; Dentsply Sirona, York, PA, USA) verified complete tooth removal. On postoperative day three, the animals were again anesthetized for inspecting and cleaning the surgical sites (Chlorhexamed^®^; GlaxoSmithKline Consumer Healthcare, Munich, Germany).

**Figure 1:**
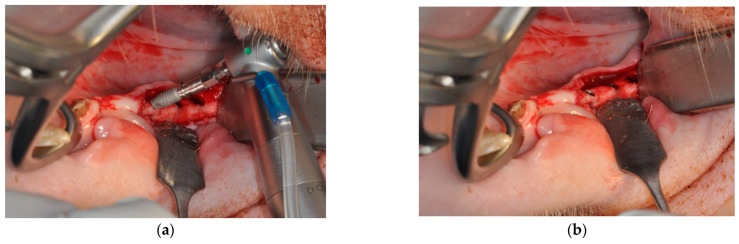
Placement of a novel implant characterized by sharp threads in the apical and cervical regions and condensing threads in the middle part of the implant (**a**). All implants were placed flush with the surface of cortical bone (**b**).

After twelve weeks of healing [2], a total of 26 dental implants were placed following local anesthesia (Ultracain D-S forte^®^), midcrestal incisions and reflection of full-thickness mucosal flaps. The implants were placed in an attempt to create a thin buccal bone wall of approximately 1 mm, with the implant shoulder being flush with the bone surface (Figure 1).

Following simple randomization by a blinded person, Control implants (n = 8; BLT^®^; SLA surface; Straumann, Basel, Switzerland), Test Porous implants (n = 7; MT^®^; sandblasted and acid-etched surface; AlfaGate, Bonn, Germany) and Test Bioactive implants (n = 9; MT^®^; calcium–phosphate surface; AlfaGate) were placed (please see Supplementary Table S1 for exact distribution), adhering to the manufacturers’ guidelines for dense bone using external irrigation with drills [7]. For measuring heat development during implant site preparation, the temperature of a 2.8 mm twist drill used in all implant groups was determined with an infrared camera immediately after removal from the osteotomy [11]. The level of underpreparation of the osteotomies was determined by measuring the final osteotomy diameter, which was then compared to the implant’s outer diameter.

**Figure 2:**
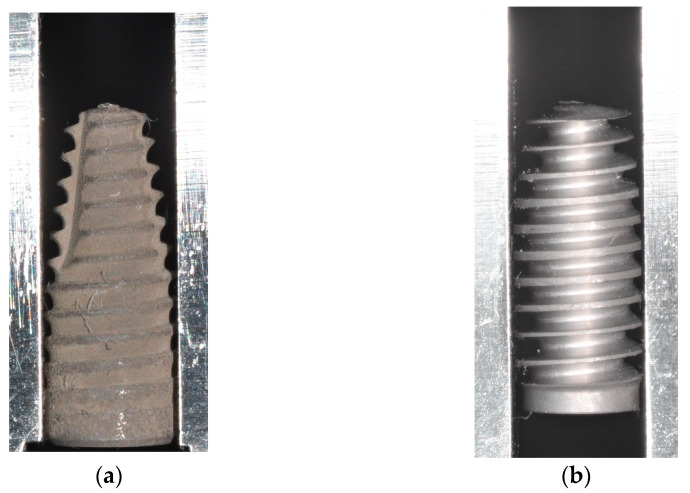
Macrodesign of the two implant types used in this study with apically tapered Control implants (**a**) and the novel implant (Test Porous, Test Bioactive) characterized by a shift in implant core diameter and thread geometry as described previously [27,28] (**b**).

Both groups of test implants had an identical macrodesign (Figure 2), but they differed in terms of surface treatment. Test Porous implants had a sandblasted and acid-etched surface with a mean roughness of R_a_ 2 µm, which was comparable to the surface of the Control implants, while Test Bioactive implants had a calcium–phosphate coating which has been characterized both in an animal study [31] and in a clinical trial [32]. Prior to the surface coating, the implants had a roughness of R_a_ 1 µm.

**Figure 3:**
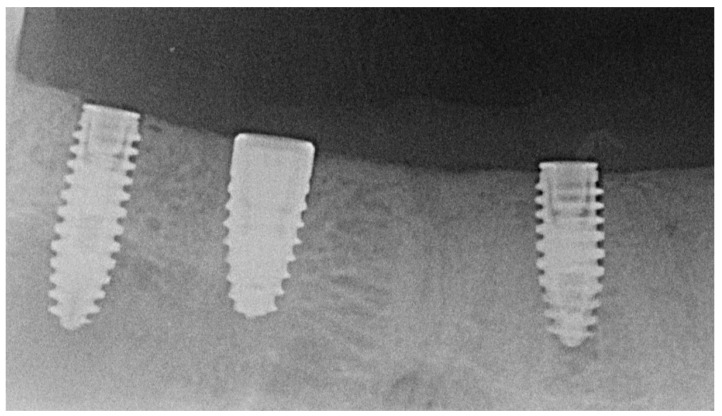
Postoperative intraoral radiograph with two test implants (right and left) and one Control implant (middle).

Primary wound closure [2] was achieved using interrupted and horizontal mattress sutures (Supramid^®^; Resorba Medical GmbH, Nürnberg, Germany) followed by intraoral radiographs (Heliodent^®^) for documenting implant positions (Figure 3). On postoperative days three and ten, the animals were again anesthetized for inspecting and cleaning the surgical sites (Chlorhexamed^®^) and for removing the sutures, respectively. The implants were allowed to heal for a total of eleven weeks.

All animals were sacrificed by an intracardial injection of T61^®^ (0.12 mL/kg body weight; Merck Animal Health, Madison, NJ, USA) following the induction of general anesthesia as described. Bone block sections containing the surgical sites were harvested and fixed in neutrally buffered formalin after all soft tissues had been removed.

**Figure 4:**
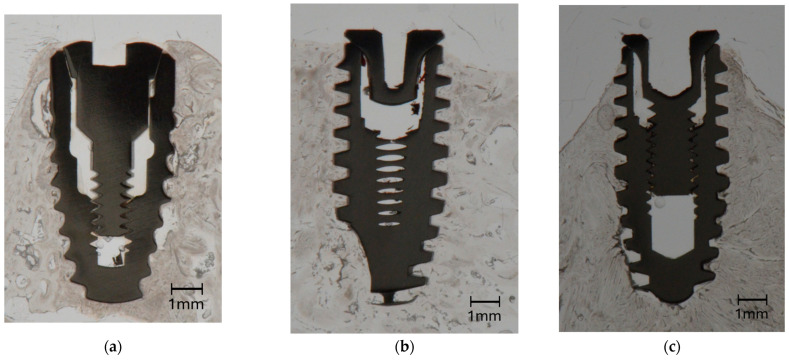
Representative unstained histologic sections showing a Control implant (**a**), a Test Porous implant (**b**) and a Test Bioactive implant (**c**) after healing.

**Figure 5:**
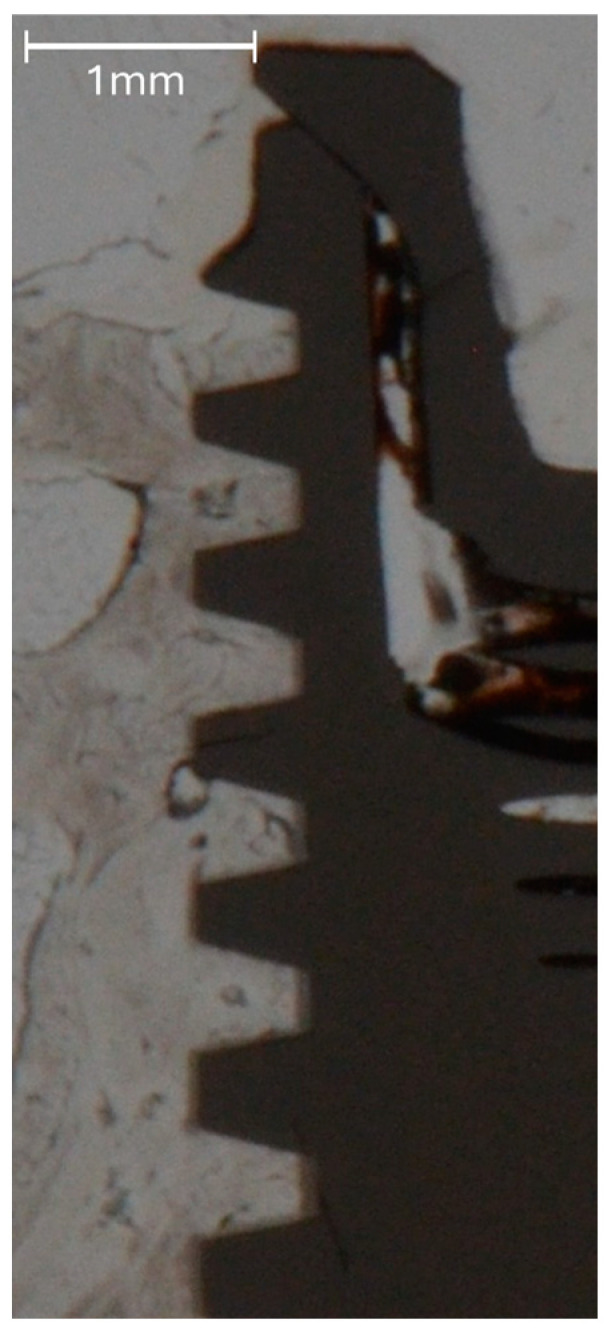
High-magnification image of the interface between bone and the implant surface for a Test Porous implant, allowing proper evaluation of BIC without staining.

The bone blocks were cut into smaller pieces, approximately 10 mm in length mesio-distally, each containing one implant, using a diamond band saw (EXAKT 300^®^; EXAKT Advanced Technologies GmbH, Norderstedt, Germany). The specimens were then dehydrated in alcohol solutions of increasing concentrations, clarified in xylene and embedded in polymethylmethacrylate (Technovit 9100^®^; Heraeus Kulzer, Hanau, Germany). One bucco-lingual section parallel to the long axis of the implant was obtained per specimen by a cutting and grinding technique [33]. With the sections reduced to a thickness of 120 µm, bone-to-implant contact (BIC) along the entire implant surface in contact with bone and marginal bone loss (MBL) as calculated by the distance between the implant shoulder and the most coronal aspect of bone in direct contact with the implant [2,13] were quantified histomorphometrically using a microscope (LEICA DM4B^®^; LEICA Mikrosysteme Vertrieb GmbH, Wetzlar, Germany) equipped with a color image analyzing system (LEICA Application Suite^®^, LEICA Phase Expert; LEICA Mikrosysteme Vertrieb GmbH). The BIC measurements were performed on images taken at a magnification of 2.5×, whereas MBL was measured on images taken at a magnification of 10× (Figure 4 and Figure 5).

Statistical analysis was based on Levene’s test on homogeneity of variances and Shapiro–Wilk tests on the normality of residuals followed by the Kruskal–Wallis rank sum test and pairwise comparisons using two-sample Wilcoxon tests with Holm correction for multiple comparisons. The level of significance was set at α = 0.05 for all statistical operations.

## 3. Results

The experimental part of this study was completed uneventfully. However, due to surgical decisions, different sample sizes were finally available for the assessment of individual parameters (Table 1). The mean values and standard deviations for all measurement values are given in Table 1. Levene’s test on homogeneity of variances did not reveal significant differences (Table 2), whereas Shapiro–Wilk tests on normality indicated significant values for all parameters with the exception of BIC (*p* = 0.60; Table 2). As a consequence, the Kruskal–Wallis rank sum test was applied. This test did not indicate any significant differences between the implant types for any parameter measured (Table 2). Similarly, the subsequently conducted pairwise two-sample Wilcoxon tests with Holm correction indicated no significant differences for any comparison (*p* > 0.05).

The drill temperatures measured immediately after finalizing the 2.8 mm osteotomy ranged between 26.0 °C for Test Porous and 27.6 °C for Control. The mean amount of undersizing of the osteotomies relative to the maximum implant diameter was 0.15 mm for Control, while 0.33 mm and 0.34 mm were used for Test Porous and Test Bioactive, respectively. Despite the greater level of undersizing, Test Porous (63.8 Ncm) reached a comparable level of insertion torque as compared to Control (61.5 Ncm). Test Bioactive implants, however, reached much greater levels of insertion torque, with a mean of 76.1 Ncm. Maximum bone-to-implant contact was observed in Test Porous implants, with 55.83%, which also showed the lowest degree of marginal bone loss, with a mean of 2.76 mm. As expected, Test Bioactive implants with the greatest insertion torques merely showed 48.11% BIC and mean MBL of 4.07 mm.

## 4. Discussion

Given that implant macrodesign seems to be a relevant parameter for early healing [13,15], this intraoral large animal study compared a novel implant design [27,28] aimed at reducing mechanical stress and trauma [2,12] in cortical bone with a well-documented conventionally tapered implant.

Osteotomies for all implant types were created by drilling, with temperature increases hardly differing between the implant systems used. Although drill design has been shown to affect heat development [7], thermal trauma [6] can be assumed to have been equal despite differences in drill design and manufacturers, which could have been seen as confounding variables. Also, the manufacturers’ standard drilling protocols [1] were not adapted to the animal situation in minipigs, characterized by very hard cortical bone [4], and only external irrigation [7] was used. The use of internal or combined external and internal irrigation [9] might have improved the histologic results due to a generally lower temperature increase. As the selection of implants with respect to diameter and length was performed intraoperatively, different lengths were present, which may be seen as a confounding variable although the region of interest was the cervical part.

The amount of undersizing of the osteotomy relative to the implant diameter was more than double in the Test implants, thereby preserving existing bone volume. Despite this, insertion torque did not differ between Control implants and Test implants (Test Porous), with a comparable surface treatment achieved by sandblasting and acid etching. The calcium–phosphate surface coating [31,32] of Test Bioactive implants led to a considerable increase in insertion torque, ultimately resulting in greater marginal bone loss. This seems to be in line with Coyac and coworkers, who showed that high insertion stress puts implants at risk for failure to osseointegrate [12]. As a consequence of this finding and being aware that osteotomy size, implant design and surface properties are the main determinants of insertion torque, a somewhat greater osteotomy size for implants with the bioactive coating seems warranted.

Despite the greater amount of undersizing, the novel implant design with the porous surface showed greater BIC and slightly lower MBL as compared to the Control implants. As such, this approach seems to be advantageous compared to implant designs not engaging buccal bone (trioval or triangular implants), which have already been shown not to improve marginal bone loss as a consequence of surgical trauma [17,18]. By switching from round implant cross-sections to triangular or trioval designs, areas of high and low peri-implant compressive strains are being generated [15], which in preclinical studies showed reduced levels of MBL [15]. The novel implant tested here [27,28] also generates areas of low and high strain but in a vertical distribution, taking advantage of soft trabecular bone being condensed more easily and undergoing less resorption while cortical bone is subject to lower levels of stress caused by sharp threads in the cervical region of the novel implant design. This favorable stress situation evoked by the novel implant has been proven to exist using strain gauge measurements in previous in vitro studies on bone surrogate materials [27,28], while this animal trial tried to link these biomechanical effects to bone response. Currently underway are additional finite element analyses, which, however, can only anticipate potential peri-implant bone loss with a distinct level of uncertainty.

The histologic measurements were made after a healing time of eleven weeks, which may be considered as being rather extended, as other authors used shorter times in order to simulate early healing [13]. However, this healing time seemed justified, as bone resorption takes some time to occur and there might have been a possibility of overlooking resorptive processes should the animals be sacrificed too early. Specific limitations of this animal trial have to be taken into consideration which are immanent in this study setup. Acknowledging the goal of reducing animal burden, the number of animals was based on comparable studies [2,7,13] without performing sample size or power calculations. As already mentioned, the bone quality was very high in this model, as also evidenced from the insertion torque values measured. For measurements of MBL, two problems have to be acknowledged, as these were purely based on histology. The implants were placed flush with the bone surface, and intraoperative X-rays were mainly performed for documentation. It would have increased the reliability of these measurements if repeated and standardized radiographs had been feasible. Also, deviations may be due to problems in determining the exact long axis of the implants during the cutting and grinding procedures. It has been shown that MBL is one of the last steps in a signaling cascade following the Wnt pathway [34], which might have been an alternative analyzing method for understanding how the implant design-related stress distribution affects bone healing in the different areas of the alveolar bone.

Of course, the insertion process is only one single aspect in the life of a dental implant, while prosthetic restoration, dynamic loading under function and patient-related factors and hygiene aspects are potentially more decisive for its long-term performance [35].

## 5. Conclusions

Following previous in vitro biomechanical testing, this animal study verified that a novel implant design deriving primary stability from compressing trabecular bone while not overstressing cortical bone and requiring less bone removal during implant site preparation performs as well as a conventional implant. Despite the promising results shown in this first animal trial, additional clinical studies are clearly needed for evaluating the potential clinical benefit of this approach.

## Figures and Tables

**Table 1:** Sample size, mean values and standard deviations for all parameters recorded in this study.

	Drill Temperature [°C]			Undersizing [mm]			Insertion Torque [Ncm]			BIC [%]			MBL [mm]		
	n	Mean	SD	n	Mean	SD	n	Mean	SD	n	Mean	SD	n	Mean	SD
**Control (Straumann BLT^®^)**	8	27.6	2.3	8	0.15	0.28	6	61.5	21.4	8	50.00	18.71	16	2.80	1.67
**Test Porous (AlfaGate MT^®^)**	7	26.0	1.0	7	0.33	0.08	6	63.8	20.8	6	55.83	23.69	14	2.76	2.07
**Test Bioactive (AlfaGate MT^®^)**	9	26.5	1.1	10	0.34	0.08	10	76.1	8.9	9	48.11	22.89	14	4.07	3.35

**Table 2:** Results (*p*-values) of statistical operations conducted. Significant differences (*p* < 0.05) are marked with *.

	Drill Temperature	Undersizing	Insertion Torque	BIC	MBL
**Levené**	0.82	0.31	0.16	0.91	0.09
**Shapiro**	0.000 *	0.000 *	0.001 *	0.600	0.009 *
**Kruskal–Wallis**	0.08	0.09	0.70	0.60	0.40

## Data Availability

The original contributions presented in this study are included in the article; further inquiries can be directed to the corresponding author.

## Supplementary Materials

The following supporting information can be downloaded at: https://www.mdpi.com/article/10.3390/jfb16030102/s1, Table S1: Distribution of implants in minipigs.

## Abbreviations

The following abbreviations are used in this manuscript:

MBL	marginal bone loss
BIC	bone-to-implant contact
