# Supplementary material for: Preclinical Performance of a Novel Dental Implant Design Reducing Mechanical Stress in Cortical Bone
Source: J Funct Biomater. 2025 Mar 14;16(3):102. doi: 10.3390/jfb16030102 (PMC11942938; doi:10.3390/jfb16030102)
Supplement: Supplementary file 1 [file jfb-16-00102-s001.zip › jfb-3472663-supplementary.pdf]

**Supplementary Table S1: Distribution of implants in minipigs**			
**Control Straumann BLT**	**Minipig**	**Position**	**Implant**
	1	45	Straumann 4.1/8
	2	34	Straumann 4.1/8
	2	45	Straumann 4.1/8
	3	36	Straumann 4.1/8
	3	46	Straumann 4.1/8
	4	36	Straumann 4.1/8
	5	36	Straumann 4.1/8
	5	46	Straumann 4.1/8
**Test Porous**	**Minipig**	**Position**	**Implant**
	1	46	Porous 4.3/10
	2	46	Porous 3.5/10
	3	34	Porous 3.5/8
	3	44	Porous 3.5/8
	4	35	Porous 3.5/8
	5	34	Porous 3.5/8
	5	45	Porous 3.5/8
**Test Bioactive**	**Minipig**	**Position**	**Implant**
	1	36	Bioactive 4.3/10
	1	34	Bioactive 4.3/10
	2	36	Bioactive 3.5/10
	2	35	Bioactive 3.5/10
	2	44	Bioactive 3.5/8
	3	35	Bioactive 3.5/8
	4	34	Bioactive 3.5/8
	4	43	Bioactive 3.5/8
	5	44	Bioactive 3.5/8
